# Communicative Strengths in Severe Aphasia: The Famous People Protocol and Its Value in Planning Treatment

**DOI:** 10.1044/2019_AJSLP-18-0283

**Published:** 2019-05-21

**Authors:** Audrey Holland, Margaret Forbes, Davida Fromm, Brian MacWhinney

**Affiliations:** a Department of Speech, Language, and Hearing Sciences, The University of Arizona, Tucson; b Department of Psychology, Carnegie Mellon University, Pittsburgh, PA

## Abstract

**Purpose:**

This clinical focus article describes the development and use of the Famous People Protocol (FPP), a clinical tool for observing the strategies people with severe aphasia (PWSA) can use to communicate when speech is limited. Its goal is to provide a systematic approach to identifying individually appropriate communication strategies for PWSA.

**Method:**

Though not a test, the FPP's development and pilot testing were consonant with qualitative approaches to test development. Eighty-one people with aphasia and 37 nonaphasic participants were given the current version of FPP and the Western Aphasia Battery–Revised (WAB-R; Kertesz, 2006). This clinical focus article reports on the 36 PWA who scored near or below the mean WAB score of the larger group.

**Results:**

The FPP has a maximum score of 100 based on (a) identification of famous people in different categories, entertainers, athletes, U.S. presidents, sports figures, and internationally famous people, and (b) responses to additional questions about the famous people. Identification is scored quantitatively on a 3-point scale, and question responses are scored correct (1) or incorrect (0). Mean scores for the PWSA and control groups were 54.6 and 95.2, respectively. FPP and WAB-R scores were moderately correlated (*r* = .67). Qualitative results describe the variety of strategies that PWSA used on the FPP.

**Conclusions:**

The FPP is a way for clinicians to engage PWSA in an activity that can reveal personally relevant strategies to help PWSA communicate more effectively. The strategies can then become the basis for subsequent training on using them conversationally. Appendixes provide examples of clinical approaches.

Remarkably few publications have examined the evaluation and treatment of people with severe aphasia (PWSA). For purposes of this clinical focus article, *severe aphasia* refers to the extent of impairment, and in addition to those with global aphasia, our sample includes a substantial number of those with Wernicke aphasia, conduction aphasia, transcortical syndromes, and Broca aphasia.

Impairment-focused instruments such as the Western Aphasia Battery–Revised (WAB-R; Kertesz, 2006) and the Boston Diagnostic Aphasia Examination–Third Edition (Goodglass, Kaplan, & Baressi, 2000) are designed to assess the many and often profound deficits that PWSA might exhibit. Although the test results effectively specify how PWSA fail in communication, they reveal little about how PWSA might achieve communicative success. Moreover, the test results offer few guidelines to clinicians about how to design effective treatments for severe aphasia, beyond the implicit suggestion that clinical interventions should attempt to reduce the deficits.

Specific intervention approaches for PWSA are relatively few. One exception is the work by Helm-Estabrooks and her colleagues (summarized in Helm-Estabrooks, Albert, & Nicholas, 2013). These researcher-clinicians have developed thoughtful rationales for training some nonverbal modalities (e.g., drawing, intoning, gesturing) in severe aphasia. Another exception is Promoting Aphasics' Communicative Effectiveness (Davis & Wilcox, 1985). This work focuses on conveying messages using whatever modalities are most effective. Several others have also considered communication strategies. Purdy and Koch (2006) focus on strategy measurement and the influence of cognitive flexibility on multimodal strategy use in their alternative scoring system for Communication Activities of Daily Living–Third Edition (Holland, Fromm, & Wozniak, 2017). Purdy and Van Dyke (2011) conducted a small-scale study of strategy training, as did Wallace, Purdy, and Skidmore (2014). Lasker and Garrett (2006) developed a promising inventory to guide the individualized selection of communication strategies for people with aphasia. None of these studies, however, provides guidelines for treatment beyond drilling strategy use in formal therapy. Crucially, none of these strategy studies presents methods for improving the functional communication of PWSA.

The goal of this project was to develop a means for discovering effective, personally relevant communication strategies for PWSA. Many years ago, Whitney (1975) called such strategies *production strategies*. When strategies such as these enhance communication and “work” for people with aphasia, they can be considered as communicative strengths that effectively get their messages across.

Positive psychology (e.g., Peterson & Seligman, 2004; Seligman, 2002, 2011) provides an appropriate rationale for strength-based training. In his presidential address to the American Psychological Association, Seligman (1999) contended that psychology as a discipline was disproportionately focused on what was “wrong” with people. To develop a balanced picture, he argued, more research effort should be focused on what is “right” with them. This became the impetus for the field of positive psychology. Our discipline is beginning to produce research that draws its motivation and rationale from positive psychology (Beck & Verticchio, 2018; Beck, Verticchio, Seeman, Milliken, & Schaab, 2017; Sather, Howe, Nelson, & Lagerwey, 2017). Positive psychologists maintain that a person's well-being and flourishing are better served by activities that support strengths than by those focused solely on overcoming deficits. Therefore, one of the clinician's roles should be to encourage PWSA to use their spontaneously occurring communication strategies more productively. In fact, these strategies can be useful across the continuum of aphasia severity.

## The Famous People Protocol

The goal of the Famous People Protocol (FPP) is to identify promising but often unrecognized strategies that individuals with aphasia can use to improve their everyday communication despite aphasia. These strategies may include writing, speaking, gesturing, drawing, or singing, or any combination of these modalities. Written and spoken responses might include circumlocutions that provide the listener with enough information to ascertain what the aphasic speaker means or simply to guess and perhaps provide a searched-for word, thus facilitating communication. Nonverbal gestures, drawing, or humming might permit the listener to recognize the intended word or concept. Aphasic speakers may sometimes request help to clarify a slightly off-target message by encouraging their listeners to guess. Once an individual's production strategies are identified, clinicians can provide training and support for the PWSA in using them. Clinicians can also encourage families to use the strategies and to prompt their aphasic family member to use them, particularly in conversational contexts. In a sense, the approach we suggest here is similar to supported communication (Kagan, 1995, 1998; Kagan, Black, Duchan, Simmons-Mackie, & Square, 2001), except that it focuses mainly on PWSA, rather than on their communication partners. We believe that most PWSA can benefit from practicing and learning to use a variety of production strategies. However, because not all strategies work for a given PWSA, they need to be carefully chosen to meet individual needs, preferences, and abilities.

These features align nicely with dynamic principles, as described by Fuerstein, Fuerstein, Falk, and Rand (2002) and elaborated upon by Hersh et al. (2013) in relation to assessment of adults with aphasia. In this more structured approach, we have not embedded strategies into ongoing language learning tasks, as suggested by Gutiérrez-Clellen and Penña (2001) and Hasson and Joffe (2007) in their work with children with language disorder. However, like dynamic assessment, the FPP provides PWSA and their clinicians with information about the strategies that might be stressed clinically as techniques for “getting messages across.”

The FPP is a charades-like activity, although it differs from charades in that PWSA are also encouraged to talk. They are specifically encouraged to use “anything they can” to identify pictures of famous people. The person with aphasia is asked to demonstrate “knowing” who the pictured person is, even if he or she cannot produce the name. This task is attractive for a number of reasons. Naming people is known to be difficult, not only for adults with language and cognitive problems (Beeson, Holland, & Murray, 1997; Gefen et al., 2013; Semenza, Mondini, Borgo, Pasini, & Sgaramella, 2003) but also for most normally aging persons (Burke & Shafto, 2008; Cohen & Burke, 1993; Evard, 2002). Thus, both PWSA and their nonaphasic partners might have difficulty in producing the names of the people pictured in the FPP. However, famous people's names are embedded in rich association networks. These networks are useful in conversation, have emotional associations, and are often personally relevant. People with aphasia tell us this when they comment that they “know it but can't say it.” This is almost the aphasic speaker's mantra. The difficulty of the task can serve as a stimulus for demonstrating the value of using alternative ways to convey messages in everyday communication. Also, retrieval of object names is relatively uninteresting, whereas naming people is fun and, for the most part, unthreatening. This clinical focus article concerns the development of the FPP and focuses on the use of FPP with persons whose aphasia is severe. The Discussion section describes some features we have observed while using the measure. The final section includes some concrete ways to apply these strategies to everyday communication

## Method

### Development and Pilot Work

The first task in developing the FPP was to choose famous people who were easily recognized by nonaphasic people of approximately the same age as most individuals with aphasia. Nonaphasic neighbors of the first author and over-60-year-old spouses of PWSA were asked to identify photographs of approximately 100 persons. All images were available on Google images; most were in color. Participants were encouraged to comment on the familiarity of the pictures and to suggest candidates for elimination. If two participants did not correctly name the person in the photograph, the photograph was dropped. That left 80 photographs from which the authors selected the final set of 24 pictures. The final stimulus set comprised 10 American (but largely world recognized) entertainers, four internationally famous persons, five American athletes representing five sports, and five U.S. presidents. Pilot testing was then conducted with 19 PWSA (14 men, five women) from 36 to 81 years of age to ensure that the administration and scoring worked and that the FPP achieved its intended goals.

The FPP uses the task of identifying famous people to encourage PWSA to “do anything they can” to communicate who the pictured people are. It is not designed to be a formal aphasia test. Rather, the primary goal of the FPP is to help clinicians, PWSA, and families to recognize what strategies work communicatively for a specific aphasic individual.

The clinician begins the FPP administration by describing the task in accordance with the guidelines of supported communication (Kagan, 1998). All instructions and the protocol items are presented in written, spoken, and pictured forms on a series of slides. For practice, a slide picturing John Wayne is presented. The clinician describes the various ways a speaker might transmit knowledge about John Wayne (naming him, calling him “The Duke,” naming a movie that he was in, gesturing him shooting a gun, etc.). To maximize comprehension, the written and spoken instructions and practice material are presented simultaneously. That is, the clinician slowly reads each item aloud while also presenting it visually. This “aphasia-friendly” format is used throughout the protocol.

To provide the clinician with additional opportunities to observe the strategies that PWSA might use to communicate effectively, 28 additional questions about the famous people were interspersed in the protocol. They are also described below.

### Scoring Guidelines

#### Famous Person Identification

The FPP is scored on a 3-point scale. A 3-point response clearly indicates that the speaker “knows” who the pictured person is, as demonstrated by providing at least two key identifying facts. For example, for Michael Jackson “moonwalk, nose job”; “Michael singer, dancer”; or simply “Michael Jackson” would all be scored as 3. Encouragers by the clinician are permitted. Vague or incomplete responses that are “in the ballpark,” such as drawing a rainbow for a picture of Judy Garland dressed as Dorothy from “the Wizard of Oz” or describing Jay Leno as “talk show,” are scored as 2. If the PWSA does not score at least 2 points, three yes/no comprehension questions are asked. For Elizabeth Taylor, for example, the questions are as follows: Did she star in Cleopatra? Did she have a lot of husbands? Is she Elizabeth Taylor? If a participant answers all three correctly, the item is scored 1; if the participant answers two or fewer correctly, the item is scored 0. Before moving on to the next item, the clinician says the correct name in an effort to avoid tip-of-the tongue frustration.

#### Additional Items

As noted above, 28 additional items were added to the protocol. They were designed to provide clinicians with more information about the communication skills and strategies that have potential for enhancing an aphasic person's communication. Most of these items are presented at the end of each subset of pictures (i.e., entertainers, world figures, sports figures, U.S. presidents). They provide opportunities to communicate by humming or singing, gesturing or pointing, or by appropriate talking. These items are presented to all PWSA regardless of their scores on the picture identification items. Each item is scored as either 1 point (*correct*) or 0 (*incorrect*). For example, after five sports figures (Tiger Woods, Peyton Manning, Mohammed Ali, Michael Jordan, the Williams sisters) are shown individually, a composite of all five is presented. The first prompt is “show me the sport that each one plays.” Another example of an additional question prompt for the sports group is: Who said, “float like a butterfly, sting like a bee?” Each correct response (gestured, spoken, written, or drawn) is scored 1 point. The total of the correct answers on these items is part of the final total score for the protocol.

#### Total Score

The final score (maximum = 100) comprises the scores (0–3) on the 24-item picture identification task (maximum = 72) plus the scores (0–1) on the 28 additional questions (maximum = 28).

### Participants

The final version of the FPP was administered to 81 PWSA from 12 aphasia centers in North America. All were seen as part of their participation in providing data for AphasiaBank (MacWhinney, Fromm, Forbes, & Holland, 2011).This clinical focus article discusses only those whose scores on the WAB-R (Kertesz, 2006) were near or below the mean of the total group, thereby including only the more severely impaired individuals. This PWSA group also includes three whose aphasia was too severe for WAB-R testing, but whose scores would have been below the mean had testing been completed (see Appendix A).

The PWSA group included 30 men and six women, with a mean age of 63.8 years (*SD* = 10.7) and a mean education level of 14.2 years (*SD* = 2.5). The mean WAB-R Aphasia Quotient (AQ) for those tested was 36.2 (*SD* = 15.3), and their aphasia types were as follows: 23 Broca, five global, two conduction, two Wernicke, and one transcortical motor. Three were classified “untestable by WAB.” Examiners explained the protocol to all participants, and all signed a consent form approved by their facility or by Carnegie Mellon University. The final version of the FPP was also administered to 37 nonaphasic participants (17 men, 20 women) ranging in age from 18 to 79 years. All but three scored 90 or above. This group included nonaphasic spouses of PWSA and nonaphasic volunteers at the testing sites. Because we wanted to ensure that the measure would be appropriate for younger PWSA, we deliberately skewed this group to include younger participants. These participants included college students and community dwellers. Graduate students in Communication Sciences and Disorders at the University of South Carolina administered the FPP to these participants as part of their training. No video recordings were made of control subjects.

## Results

### Quantitative Analysis

**Figure 1.**
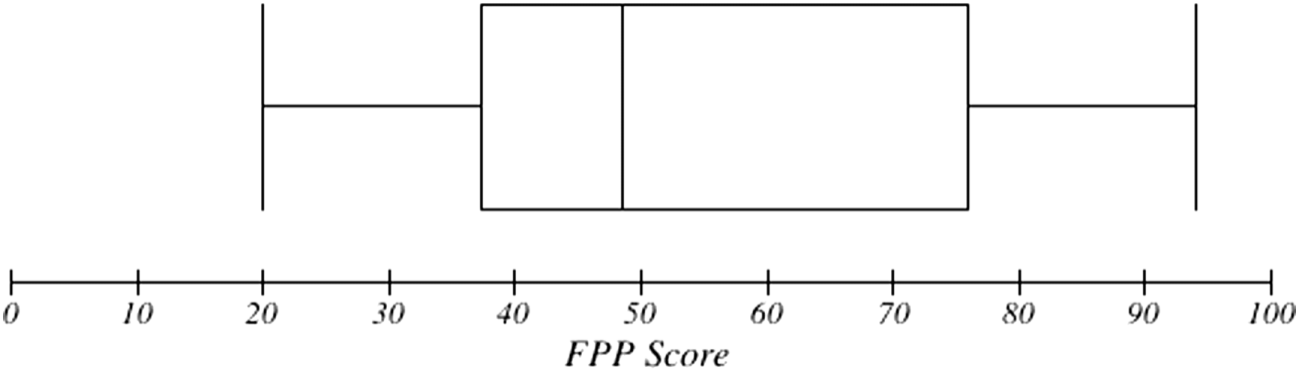
Box plot of Famous People Protocol (FPP) scores for the people with severe aphasia group.

**Figure 2.**
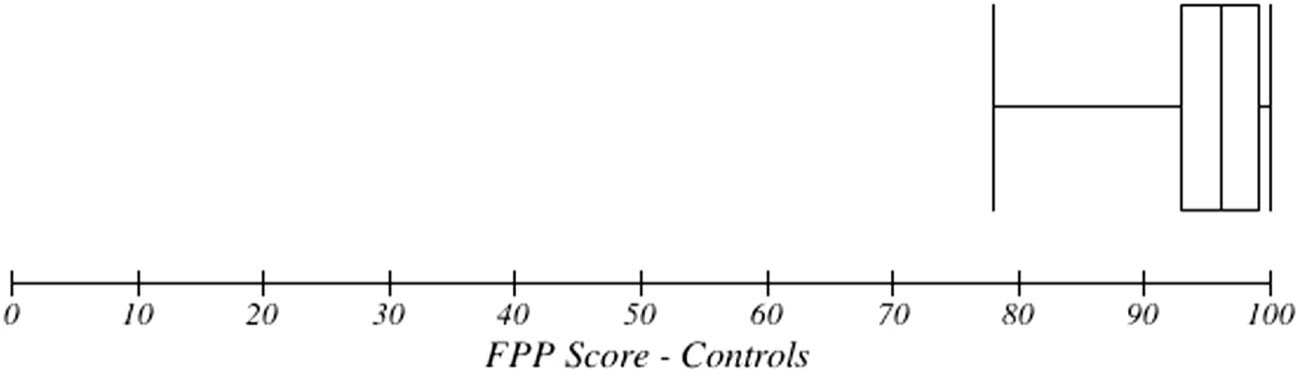
Box plot of Famous People Protocol (FPP) scores for the control group.

The mean FPP score for the entire PWSA group on FPP was 54.6 (*SD* = 21, range: 20–94). The box plot in Figure 1 shows the distribution of scores, with the minimum, maximum, first and third quartiles, and median score. The mean score for the control group was 95.2 (*SD* = 4.8, range: 78–100). The box plot in Figure 2 shows them to be normally distributed but across a much more restricted range of scores.

### Relevant Correlations

We expected that scores on the FPP would show a partial correlation with scores in the WAB-R AQ. This correlation should not be perfect; the WAB-R does not measure the PWSA's ability to access residual naming strategies. In fact, the Pearson product–moment correlation between the FPP and the WAB-R AQ was significant (*r* = .67, *p* < .05) but moderate. Also, age and the FPP were significantly correlated (*r* =.43, *p* < .05). This result reflects a tendency for older individuals to do better on this measure. The FPP and education (in years) were not significantly correlated (*r* = .08). For the control group, FPP was not significantly correlated with age (*r* = .26) or education (*r* = .23).

### Reliability

Two of the authors (A. L. H. and M. F.) watched videos of FPP administrations for 10 randomly selected participants and independently did qualitative coding for the 24 pictures that could be correct (scored as 3) or in the ballpark (scored as 2). Point-by-point intercoder agreement across the items scored was 89%.

## Discussion

The modest but statistically significant correlation of the FPP with the WAB-R AQ indicates that the FPP provides relevant information that is qualitatively different from that provided by the WAB-R for a more severely impaired sample. The naming section of the WAB-R and other impairment-focused inventories typically assess only whether the person provides the right answer (or which of a limited set of cues facilitates the “right” answer). Such tests do not attempt to elicit, record, or credit other aspects of the individual's knowledge or communication abilities. Thus, we believe the FPP provides additional, meaningful, and clinically helpful information that standard tests do not provide. In effect, the FPP provides information about the extent to which certain aspects of functional communication are preserved, despite the aphasia-producing brain damage. Because the FPP uses familiar stimuli and allows participants to use all forms of functional communication, it provides a more complete picture of residual communication abilities than traditional confrontation naming assessments. We believe it is crucial for clinicians to consider world knowledge and experience and encourage PWSA to share what they know using all available communicative means as a way to look beyond the impairment of aphasia to positive methods for everyday communication. The FPP allows PWSA to explore and demonstrate the strategies they can use to get their messages across. Once PWSA have demonstrated which communicative strategies are available and useful, their families and clinicians can encourage them to use these strengths in everyday communication. Such information was welcomed by the majority of participants in this study.

Traditional clinical approaches, such as practicing words—particularly personally relevant vocabularies—have value; we suggest that encouraging the use of personally relevant strategies should also be embedded in treatment. Our results suggest that there is a sense of personal familiarity with famous people that provides some of the necessary emotional valence or hook.^1^

It should be noted that even when PWSA in our sample could not name a person, they still overwhelmingly preferred to attempt verbal strategies, either alone or in combination with another modality (see Appendix B for examples). This suggests that, when PWSA have trouble getting their messages across effectively, it might be necessary to model and encourage the use of combinations, such as writing or gesturing in combination with cued speech. This seems particularly important for individuals whose aphasia is accompanied by apraxia of speech.

Although we were careful to select famous people for FPP stimuli, we know that, in some instances, fame is durable (e.g., Abraham Lincoln), but in other instances, such as sports, it may be fleeting (e.g., Peyton Manning should now be replaced by Tom Brady). We intend to update the current FPP within the next few years, and clinicians are encouraged to substitute regionally relevant famous people.^2^

We are not proposing this task for formal psychometric assessment or standardization. Rather, it is a clinical tool for exploring PWSA's communication abilities and strategies. Quantitative and qualitative scoring of the protocol or selected parts of the protocol may provide informative benchmarks for a given individual over time. Participants' age did not appear to be a significant issue. Our youngest control subject was 18 years old when tested, and his score was 96. We were also concerned that cultural issues might be pertinent. Would the FPP be culturally appropriate for Americans for whom English was their second (or third) language? Although only eight were studied, it is notable that one of the early pilot subjects had been in the United States for only 10 years before his aphasia-producing stroke. Prestroke, he was fluent in Amharic, French, and English. He scored 90 on the pilot version of the FPP.

We were also fortunate to have used the FPP in Halifax, Nova Scotia, at Dalhousie University's intensive aphasia treatment program (InterACT), where six participants were Canadian and three were American. They provided a window on a North American culture that differs from that of the United States. An English-speaking Canadian control participant was also studied. He achieved a perfect score. English was the first language for eight of the PWSA from Dalhousie. No cultural problems were noted, and although we worried that naming U.S. presidents could present a problem, Canadians had little difficulty. The Canadians made useful suggestions concerning changes that would make the protocol more “Canada-friendly” (all of them suggested replacing the U.S. football player with a Canadian hockey icon).

We believe that the FPP is a useful clinical adjunct to traditional testing. It is designed to help clinicians, families, and PWSA observe and develop communicative skills that remain, regardless of the severity of impairment resulting from aphasia. It provides insight into the disability and handicap that aphasia may impose on getting along in the everyday world. The FPP should give clinicians useful information about how to help PWSA and their communication partners capitalize on their strengths in everyday communication.

### Availability of FPP

Detailed instructions for administering the FPP, its scoresheet, scoring guidelines, the stimulus pictures, and aphasia-friendly instructions for administration are in the public domain of the AphasiaBank website at http://aphasia.talkbank.org/famous/. Videos of individuals with aphasia who agreed to have their videotapes viewed are also available at the website.

## Footnotes

1 For example, side comments like patting one's hand on one's heart occurred for some loved figures; dislike of a past president or world figure might be indicated with any variety of comments, gestures, and so forth.

2 For example, popular college basketball coaches in states where college basketball is revered or regionally recognized stars of different genres of music.

## Appendix A

### Three Brief Case Studies and Their Clinical Treatment Application

We chose three individuals with differing aphasic syndromes to illustrate how the Famous People Protocol (FPP) might be applied to treatment. Their numbers in the FPP database at AphasiaBank are provided so that interested clinicians can locate them and observe their FPP performances.

**Jack (scale31).** Jack scored a 51.5 on the Western Aphasia Battery–Revised (WAB-R) Aphasia Quotient 1 day before he participated in the FPP study. His aphasia type was Broca, and he was 2 years poststroke and 64 years old. The WAB-R testing was immediately followed by the AphasiaBank discourse protocol. Although he was cooperative and friendly throughout, Jack's discourse protocol video shows constant struggle behavior and minimal communicative speech. When he was asked if he would be willing to return the following day to do the FPP, surprisingly, Jack agreed. Freed of testing constraints and encouraged to get his messages across any way he could, his FPP score of 94 put him in the normal range. His performance was a combination of occasionally correct verbal responses but also by part- and whole-word writing. His wife reported later that he told her he had a “good time” with the FPP. Our belief is that the FPP afforded him a chance to be himself or, as A. Kagan (personal communication, 2017) suggests, “a chance to forget he had aphasia for a while.”

Therapy for Jack might focus on traditional, impairment-focused naming practice or on specific techniques such as semantic feature analysis for nouns (Boyle, 2015; Boyle & Coelho, 1995) or verb network strengthening treatment (Edmonds & Babb, 2011; Edmonds, Mammino, & Ojeda, 2014). Because he can write well, Jack should be encouraged to use writing to accompany his speaking attempts. This strategy can be added to almost any clinical treatment approach. For example, if Jack's impairment-based treatment uses semantic feature analysis and if the target word is *bread,* his clinician could easily encourage Jack to write bread's features by reminding him: “Writing it is as good as saying it. So, if you want a piece and **can't** say it, you **can** write it!” Our opinion is that Jack should also be encouraged to circumlocute or to “try his speech OR writing wings” with approaches such as “Okay, you can't say bread, but writing will get you halfway to a sandwich” or “If you were really hungry, what other words (written or spoken) could help you get a slice of it?”

In essence, Jack (and his interlocutors) should be helped to realize the utility of using these strategies through both impairment-focused speech therapy and counseling. Thus, his clinician should point out frequently that writing is his strength, that writing is communicative, and that writing takes pressure off his interlocutors when he reverts to it. Finally, it is his path to real-world communicating again. Conversational coaching (Hopper, Holland, & Rewega, 2002) would be appropriate, incorporating his wife, to serve as a “communication coach” in addition to the clinician.

**Roger (kurland15fp).** Roger presents a quite different picture. He had two WAB-R evaluations at 6 months and 1 year poststroke. He received a consistent score of 10.8, and his aphasia was classified as Broca. He was subsequently classified as untestable. He was followed for the next 2 years as part of research protocol and was administered the FPP at the end of those 2 years. Our clinical impression was that Roger had a fluent global aphasia. He was 65 years old when he participated in the FPP study. Roger and his partner own and operate a mail-order business, and since his aphasia-producing stroke, Roger's responsibilities in the company have centered on filling orders and getting them ready for shipment.

The support provided by FPP stimuli and its instructions gave Roger a platform for demonstrating his knowledge by use of at least five strategies: singing, drawing, gesturing/pantomiming, part-word writing, and occasional part-word naming. Rather than viewing it as a test, Roger viewed the FPP as a game, and although his score of 50 was not above the mean, it was close. Clinically, although his responses suggested many avenues to work with, the bottom line was that Roger was a successful communicator and a gregarious individual, despite severe aphasia. He was doing well without clinical intervention. Nonetheless, FPP's game-like features gave Roger an opportunity to demonstrate his flexibility, and he clearly enjoyed trying every way he could to communicate. It might be worthwhile to point this out to his communication partners.

However, should more treatment be sought, his clinician should probably design activities rather like the FPP, but within explicit categories representing Roger's interest in musical theatre (Broadway shows, names of its stars, well-known songs, etc.) and travel (famous places, country names, etc.). It might be informative to Roger and to his clinician to use the same stimuli across practice focused on his different strategies (e.g., “Today, let's use just gesture and pantomime to communicate”) in successive therapy sessions.

As noted above, Roger's initial approach to the FPP was as though it were a game of charades. Indeed, charades itself could be a very useful group activity in treatment of aphasia that focuses on getting messages across in whatever way possible. The only difference from everyday charades should be to encourage speech and other modalities.

**Luis (elman21a).** According to his referring clinician, Luis was not testable with the WAB-R. He was 63.5 years old and 2 years poststroke. Prestroke, Luis had been the chief accountant for a moderate-sized company, where the predominant language was English. Luis is unique in this sample for a number of reasons: (a) He was premorbidly fluent in two languages (English and Spanish); (b) he communicated via “thumbs up/down” and has very limited use of a computer equipped with a program that he was inadequately prepared to use and that appeared to be beyond his limited skills; (c) he had only very recently started to attend the aphasia center where he was seen for this FPP administration and, therefore, was relatively unknown there; and (d) he received weekly individual treatment at another facility. During the FPP administration, it quickly became apparent that this man, who appeared to be globally aphasic, with very limited use of his left hand and a significant swallowing problem, had good auditory comprehension. In addition, having lived in the United States for 20 years, Luis was no stranger to American pop culture and obtained a score of 40 on the FPP, largely by his use of thumbs up/down along with reminders to use his computer.

Although Luis' score was below the mean for the FPP, the “untestable with the WAB-R” classification seems misleading, at best. Regardless, in our view, the next step in his treatment should be to get him to use a manageable speech-generating device, starting with a careful assessment of appropriate augmentation and some substantial consultation regarding better positioning for using it. The Multimodal Communication Screening Test for Persons With Aphasia (Lasker & Garrett, 2006) would be a valuable asset for evaluation. It should also provide information relevant to using both his auditory comprehension strengths and some appropriate technology to improve his communication.

Regarding suggested strategies, one of the most useful should be a carefully selected and managed augmentative and alternative communication approach. A strength in Luis' case is his devoted family who appeared to be eager to help him in any way. Another possible strategy is Visual Action Therapy (Helm-Estabrooks et al., 2013). We would suggest FPP-like tasks and helping him to use his device to select a correct answer. For example, the clinician might use a small stimulus set of photos arrayed in groups of four or six. She could provide clues and require Luis to select the person or place. The stimulus could be “a very important church in Rome,” and Luis would have to choose from among St. Peter's, Notre Dame, and Westminster Cathedral. In addition, given the extent of Luis' aphasia, it is likely that he has only limited opportunities to initiate either conversational topics or activities. To the greatest possible extent, the focus should be what he wants to do or would at least put up with. Suggestions can be found in Garrett and Lasker (2007) and King (2013). In view of his significant apraxia of speech, impairment work should be directed toward that problem.

## Appendix B

### Illustrative 3-Point Responses

Below are illustrative responses to FPP pictures that were scored as correct (3 points). Responses scored as 2 are variations on these types, as described previously. Responses scored as 1 are indications of comprehension of relevant yes/no answers to the FPP pictures or responses to the 28 interspersed questions.

**Table:**

Stimulus	Response type	Example
Clint Eastwood	spoken	*Clint Eastwood, Eastwood*
Judy Garland	circumlocution	*She's the one with those Toto guys*
The Beatles	conduit d'approche	*bangles, bingles, bungles, Beatles*
Marilyn Monroe	singing, humming	*Happy Birthday, Mr. President*
Marilyn Monroe	gestural	panty-revealing swirling skirt from the film “7-Year Itch”
Adolph Hitler	drawing	draw swastika
Adolph Hitler	speech plus gesture	*Heil* and Nazi salute
Willy Nelson	spoken	*country singer*
Willy Nelson	gesture	long hair
George Washington	spoken	*the first one*
Michael Jordan	spoken	*Bulls*
Michael Jordan	gesture	shooting basketball
Willie Nelson	cued spoken	cue: *Willie* response: *Netson*
Osama bin Laden	cued	cue: /ou/ response: *dead*
